# Online misinformation is linked to early COVID-19 vaccination hesitancy and refusal

**DOI:** 10.1038/s41598-022-10070-w

**Published:** 2022-04-26

**Authors:** Francesco Pierri, Brea L. Perry, Matthew R. DeVerna, Kai-Cheng Yang, Alessandro Flammini, Filippo Menczer, John Bryden

**Affiliations:** 1 Dipartimento Di Elettronica, Informazione E Bioingegneria, Politecnico Di Milano, Milano, Italy; 2 Department of Sociology, Indiana University, Bloomington, IN USA; 3 Observatory On Social Media, Indiana University, Bloomington, IN USA

**Keywords:** Epidemiology, Information technology

## Abstract

Widespread uptake of vaccines is necessary to achieve herd immunity. However, uptake rates have varied across U.S. states during the first six months of the COVID-19 vaccination program. Misbeliefs may play an important role in vaccine hesitancy, and there is a need to understand relationships between misinformation, beliefs, behaviors, and health outcomes. Here we investigate the extent to which COVID-19 vaccination rates and vaccine hesitancy are associated with levels of online misinformation about vaccines. We also look for evidence of directionality from online misinformation to vaccine hesitancy. We find a negative relationship between misinformation and vaccination uptake rates. Online misinformation is also correlated with vaccine hesitancy rates taken from survey data. Associations between vaccine outcomes and misinformation remain significant when accounting for political as well as demographic and socioeconomic factors. While vaccine hesitancy is strongly associated with Republican vote share, we observe that the effect of online misinformation on hesitancy is strongest across Democratic rather than Republican counties. Granger causality analysis shows evidence for a directional relationship from online misinformation to vaccine hesitancy. Our results support a need for interventions that address misbeliefs, allowing individuals to make better-informed health decisions.

## Introduction

The COVID-19 pandemic has killed over 4.9 million people and infected 241 million worldwide as of October 2021^1^. Vaccination is the lynchpin of the global strategy to fight the SARS-CoV-2 coronavirus^2,3^. Surveys conducted during February and March 2021 found high levels of vaccine hesitancy with around 40–47% of American adults hesitant to take the COVID-19 vaccine^4,5^. However, populations must reach a threshold vaccination rate to achieve herd immunity (i.e., 60–70%)^6–8^. Evidence of uneven distributions of vaccinations^9^ raises the possibility of geographical clusters of non-vaccinated people^10^. In early July 2021, increased rates of the highly transmissible SARS-CoV-2 Delta variant were recorded in several poorly vaccinated U.S. states^9^. These localized outbreaks will preclude eradication of the virus and may exacerbate racial, ethnic, and socioeconomic health disparities.

Vaccine hesitancy covers a spectrum of intentions, from delaying vaccination to outright refusal to be vaccinated^11^. Some factors are linked to COVID-19 vaccine hesitancy, with rates in the U.S. highest among three groups: African Americans, women, and conservatives^12^. Other predictors, including education, employment, and income are also associated with hesitancy^13^. A number of studies discuss the spread of vaccine misinformation on social media^14^ and argue that such campaigns have driven negative opinions about vaccines and even contributed to the resurgence of measles^15,16^. In the COVID-19 pandemic scenario, widely shared misinformation includes false claims that vaccines genetically manipulate the population or contain microchips that interact with 5G networks^17,18^. Exposure to online misinformation has been linked to increased health risks^19^ and vaccine hesitancy^20^. Gaps remain in our understanding of how vaccine misinformation is linked to broad-scale patterns of COVID-19 vaccine uptake rates.

The Pfizer-BioNTec COVID-19 vaccine was the first to be given U.S. Food and Drug Administration Emergency Use Authorization (EUA) on December 10th 2020^21^. EUA was then given to two other vaccines in early 2021. Initially, vaccines were selectively administered with nationwide priority being given to more vulnerable cohorts such as elderly members of the population. As vaccines became available to the entire adult population^22^, adoption was driven by limits in demand rather than in supply. It is therefore important to study the variability in uptake across U.S. states and counties, as reflected in recent surveys^23,24^.

Here we study relationships between vaccine uptake, vaccine hesitancy, and online misinformation. Leveraging data from Twitter, Facebook, and the Centers for Disease Control and Prevention (CDC), we investigate how online misinformation is associated with vaccination rates and levels of vaccine hesitancy across the U.S. We also use Granger Causality analysis to investigate whether there is evidence for a directional association between misinformation and vaccine hesitancy.

## Methods

Our key independent variable is the mean percentage of vaccine-related misinformation shared via Twitter at the U.S. state or county level. We used 55 M tweets from the CoVaxxy dataset^17^, which were collected between January 4th and March 25th from the Twitter filtered stream API using a comprehensive list of keywords related to vaccines (see Supplementary Information). We leveraged the Carmen library^29^ to geolocate almost 1.67 M users residing in 50 U.S. states, and a subset of approximately 1.15 M users residing in over 1,300 counties. The larger set of users accounts for a total of 11 M shared tweets. Following a consolidated approach in the literature^25–28^, we identified misinformation by considering tweets that contained links to news articles from a list of low-credibility websites compiled by a politically neutral third party (see details in the Supplementary Information). We measured the prevalence of misinformation about vaccines in each region by (i) calculating the proportion of vaccine-related misinformation tweets shared by each geo-located account; and (ii) taking the average of this proportion across accounts within a specific region. The Twitter data collection was evaluated and deemed exempt from review by the Indiana University IRB (protocol 1102004860).

Our dependent variables include vaccination uptake rates at the state level and vaccine hesitancy at the state and county levels. Vaccination uptake is measured from the number of daily vaccinations administered in each state during the week of 19–25 March 2021, and measurements are derived from the CDC^9^. Vaccine hesitancy rates are based on Facebook Symptom Surveys provided by the Delphi Group^24^ at Carnegie Mellon University. Vaccine hesitancy is likely to affect uptake rates, so we specify a longer time window to measure this variable, i.e., the period January 4th–March 25th 2021. We computed hesitancy by inverting the proportion of individuals “who either have already received a COVID vaccine or would definitely or probably choose to get vaccinated, if a vaccine were offered to them today.” See Supplementary Information for further details.

There are no missing vaccine-hesitancy survey data at the state level. Observations are missing at the county level because Facebook survey data are available only when the number of respondents is at least 100. We use the same threshold on the minimum number of Twitter accounts geolocated in each county, resulting in a sample size of N = 548 counties.

Our multivariate regression models adjust for six potential confounding factors: percentage of the population below the poverty line, percentage aged 65 + , percentage of residents in each racial and ethnic group (Asian, Black, Native American, and Hispanic; White non-Hispanic is omitted), rural–urban continuum code (RUCC, county level only), number of COVID-19 deaths per thousand, and percentage Republican vote (in 10 percent units). Other covariates, including religiosity, unemployment rate, and population density, were also considered (full list in Supplementary Table S9).

We also conduct a large number of sensitivity analyses, including different specifications of the misinformation variable (with a restricted set of keywords and different thresholds for the inclusion of Twitter accounts) as well as logged versions of misinformation (to correct positive skew). These results are presented in Supplementary Information (Tables S3-S8).

We conduct multiple regression models predicting vaccination rate and vaccine hesitancy. Both dependent variables are normally distributed, making weighted least squares regression the appropriate model. Data are observed (aggregated) at the state or county level rather than at the individual level. Analytic weights are applied to give more influence to observations calculated over larger samples. The weights are inversely proportional to the variance of an observation such that the variance of the *j*-th observation is assumed to be σ^2^/*w*_j_ where *w*_j_ is the weight. The weights are set equal to the size of the sample from which the average is calculated. We estimate weighted regression with the aweights command in Stata 16. In addition, because counties are nested hierarchically within states, we use cluster robust standard errors to correct for lack of independence between county-level observations.

We investigate Granger causality between vaccine hesitancy and misinformation by comparing two auto-regressive models. The first considers daily vaccine hesitancy rates $x$ at time $t$ in geographical region $r$ (state or county):

$$x_{t,r} = \mathop \sum \limits_{i}^{n} a_{i} x_{t - i,r} + \epsilon_{t,r} ,$$

where $n$ is the length of the time window. The second model adds daily misinformation rates per account as an exogenous variable $y$:

$$x_{t,r} = \mathop \sum \limits_{i}^{n} (a_{i} x_{t - i,r} + b_{i} y_{t - i,r} ) + \epsilon^{{\prime }}_{t,r} .$$

The variable $y$ is said to be Granger causal^30,31^ on $x$ if, in statistically significant terms, it reduces the error term $\epsilon^{\prime}_{t}$, i.e., if

$$E_{{a,b}} = \sum\limits_{{t,r}} {\epsilon _{{t,r}} ^{2} } - \sum\limits_{{t,r}}^{{}} {\epsilon _{{t,r}}^{{\prime 2}} } > 0,$$

meaning that misinformation rates *y* help forecast hesitancy rates *x*. We assume geographical regions to have equivalence and independence in terms of the way misinformation influences vaccine attitudes. Thus, we use the same parameters for $a_{i}$ and $b_{i}$ across all regions. We employ Ordinary Least Squares (using the Python statsmodels package version 0.11.1) linear regression to fit $a$ and $b$, standardizing the two variables and removing trends in the time series of each region. We select the value of the time window $n$ that maximizes $E_{a,b}$. For both counties and states, this was $n = 6$ days and we present results using this value. We also tested nearby values of $n \pm 2$ to confirm these provide similar results. We use data points with at least 1 tweet and at least 100 survey responses for every day in the time window for the specified region.

The traditional statistic used to assess the significance of Granger Causality is the F-statistic^30^. However, in our case, there are several reasons why this is not appropriate. First, we have missing time windows in some of our regions. Second, our assumptions of equivalence and independence for regions may not be accurate. For these reasons, we use a bootstrap method to estimate the expected random distribution of $E_{a,b}$ with the time signal removed. To this end, we generate trial surrogates for $y$ by randomly shuffling the data points. With each random reshuffled trial, we can then use the same procedure to calculate the reduction in error, which we call $E^{*}_{a,b}$. The *p*-value of our Granger Causality analysis is then given by the proportion of trials ($N$ = 10,000) for which $E^{{*}{}}_{a,b} > E_{a,b}$. A potential issue with Granger Causality analysis is that it may detect an underlying trend. We tested for this by linearly detrending both time series before running the Granger analysis, finding similar results.

## Results

**Figure 1:**
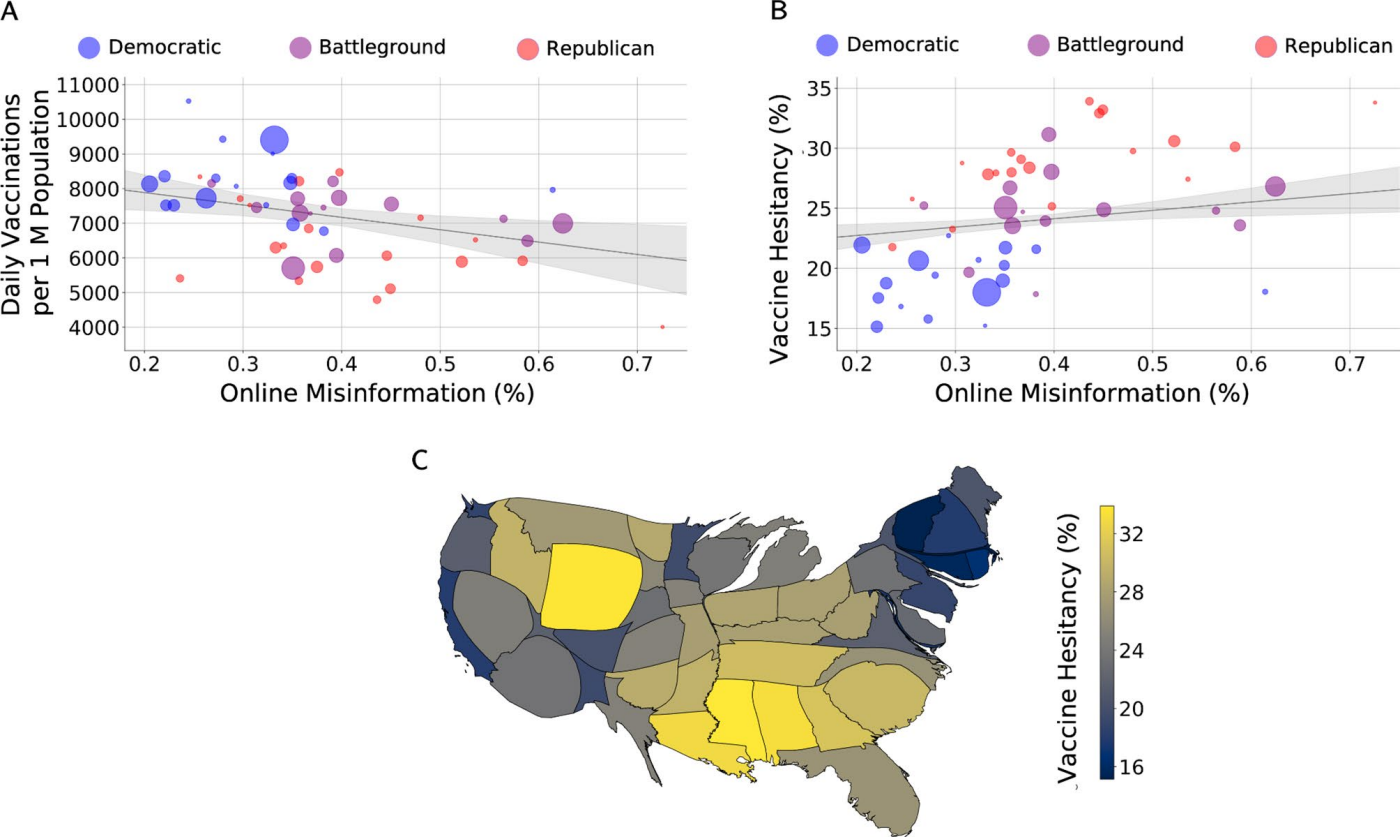
Online misinformation is associated with vaccination uptake and hesitancy at the state level. (**A**) State-level mean daily vaccinations per million population during the period from March 19 to 25, 2021, against the average proportion of vaccine misinformation tweets shared by geolocated users on Twitter during the period from January 4 to March 25, 2021. (**B**) Levels of state-wide vaccine hesitancy, computed as the fraction of individuals who would not get vaccinated according to Facebook daily surveys administered in the period from January 4 to March 25, 2021, and misinformation about vaccines shared on Twitter. Each dot represents a U.S. state and is colored according to the share of Republican voters (battleground states have a share between 45 and 55%) and sized according to population. Grey lines show the partial correlation between the two variables after adjusting for socioeconomic, demographic, and political factors in a weighted multiple linear regression model (shaded areas correspond to 95% C.I.). (**C**) Cartogram^33^ of the U.S. in which the area of each state is proportional to the average number of misinformation links shared by geolocated users, and the color is mapped to the vaccine hesitancy rate, with lighter colors corresponding to higher hesitancy (image generated by https://go-cart.io under CC-BY license).

Looking across U.S. states, we observe a negative association between vaccination uptake rates and online misinformation (Pearson *R* = –0.49, *p* < 0.001). Investigating covariates known to be associated with vaccine uptake or hesitancy, we find that an increase in the mean amount of online misinformation is significantly associated with a decrease in daily vaccination rates per million (*b* = –3518.00, *p* = 0.009, Fig. 1A, and see Methods and Supplementary Table S1). Political partisanship (a 10% increase in GOP vote) is also strongly associated with vaccination rate (*b* = –640.32, *p* = 0.004). These two factors alone explain nearly half the variation in state-level vaccination rates, and are themselves moderately correlated (Supplementary Fig. S1 and Table S1), consistent with prior research^32^. Remaining covariates are non-significant and/or collinear with other variables (i.e., have high variance inflation factors) and thus dropped for parsimony.

To investigate vaccine hesitancy, we leverage over 22 M individual responses to daily survey data provided by Facebook^24^ (see Methods). Reports of vaccine hesitancy are aggregated at the state level (given as a percentage) and weighted by sample size. We find a strong negative correlation between vaccine uptake and hesitancy across U.S. states (Pearson *R* = –0.71, *p* < 0.001, Supplementary Fig. S1), suggesting that daily vaccination rates largely reflect demand for vaccines rather than supply. Taking into account the same set of potential confounding factors in a weighted regression model, we find a significant positive association between misinformation and state-level vaccine hesitancy (*b* = 6.88, *p* = 0.007), and between political partisanship and hesitancy (*b* = 2.96, *p* < 0.001; see Fig. 1B and Supplementary Fig. S1). Figure 1C illustrates the state-level correlation between misinformation and hesitancy. For example, the large size and yellow color of Wyoming indicate it is the state with the highest level of misinformation and hesitancy. Among other variables, we find that the percentage of Black residents is positively related to reports of hesitancy (*b* = 0.12, *p* = 0.001), while the percentage of Hispanic or Latinx residents is negatively associated (*b* = –0.07, *p* = 0.021). The percentage of residents below the poverty line is also positively associated with vaccine hesitancy (*b* = 0.53, *p* = 0.001).

**Figure 2:**
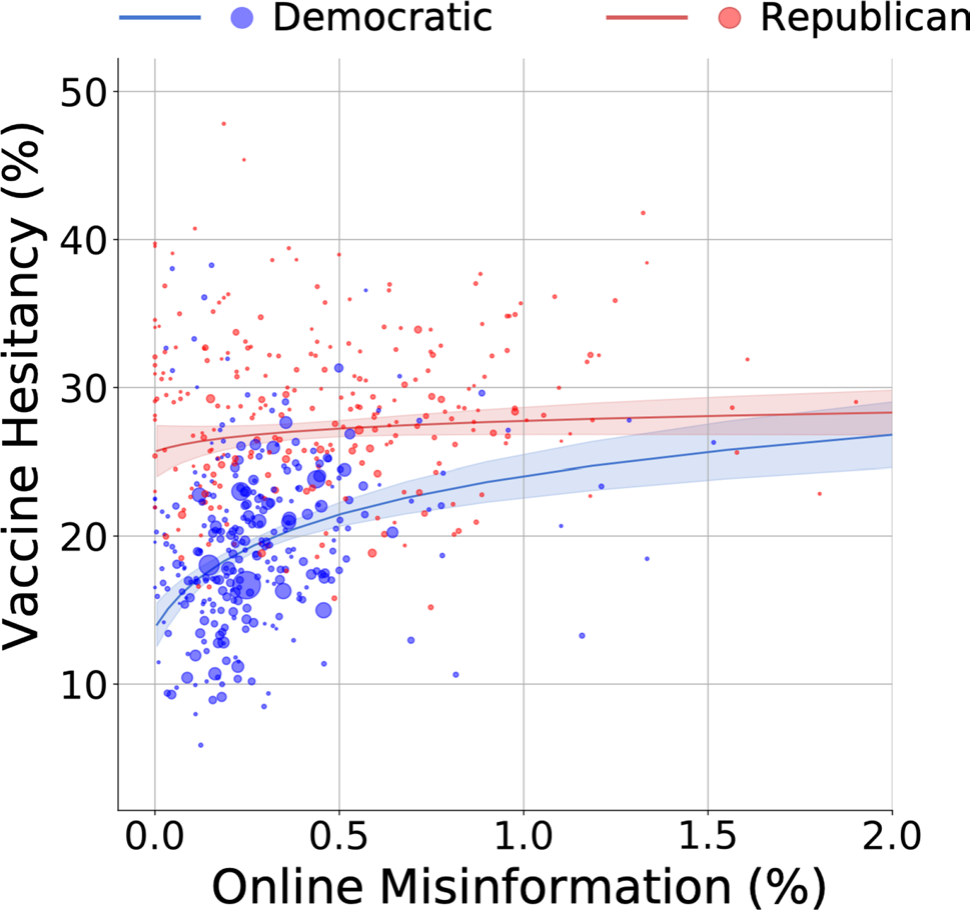
Associations of online misinformation and political partisanship with vaccination hesitancy at the U.S. county level. Each dot represents a U.S. county, with size and color indicating population size and political majority, respectively. The average proportion of misinformation shared on Twitter by geolocated users was fitted on a log scale due to non-normality (i.e., positive skew) at the county level. The two lines show predicted values of vaccine hesitancy as a function of misinformation for majority Democratic and Republican counties, adjusting for county-level confounding factors (see Methods). Shaded area corresponds to 95% C.I.

To test the robustness of these results, we also consider a more granular level of information by examining county data. Similar to previous analyses, we compute online misinformation shared by almost 1.15 M Twitter users geolocated in over 1,300 U.S. counties. We measure vaccine hesitancy rates by leveraging over 17 M daily responses to the Facebook survey for over 700 distinct counties. The total number of observations (counties) for which we are able to measure both variables is *N* = 548 (see Methods). Political partisanship and misinformation are both significantly correlated with county-level vaccine hesitancy, net covariates (Supplementary Table S4 and Supplementary Fig. S2). Using a weighted multiple linear regression model, we find a significant interaction between political partisanship and misinformation. Specifically, as levels of misinformation increase, Democratic and Republican counties converge to the same level of vaccine hesitancy (Fig. 2). This may suggest the presence of a ceiling effect at around 30% of residents being vaccine hesitant (on average), with Republican counties having already reached the ceiling and thus their residents being less likely to be affected by misinformation.

Our results so far demonstrate an association between online misinformation and vaccine hesitancy. We investigate evidence for directionality in this association by performing a Granger Causality analysis^30,31^. We find that misinformation helps forecast vaccine hesitancy, weakly at state level (*p* = 0.0519) and strongly at county level (*p* < 0.001; see Methods and Supplementary Tables S10, S11). Analysis of the significant lagged coefficients (Supplementary Table S10) indicates that there is a lag of around 2–6 days from misinformation posted in a county to a corresponding increase in vaccine hesitancy in the same county.

**Figure 3:**
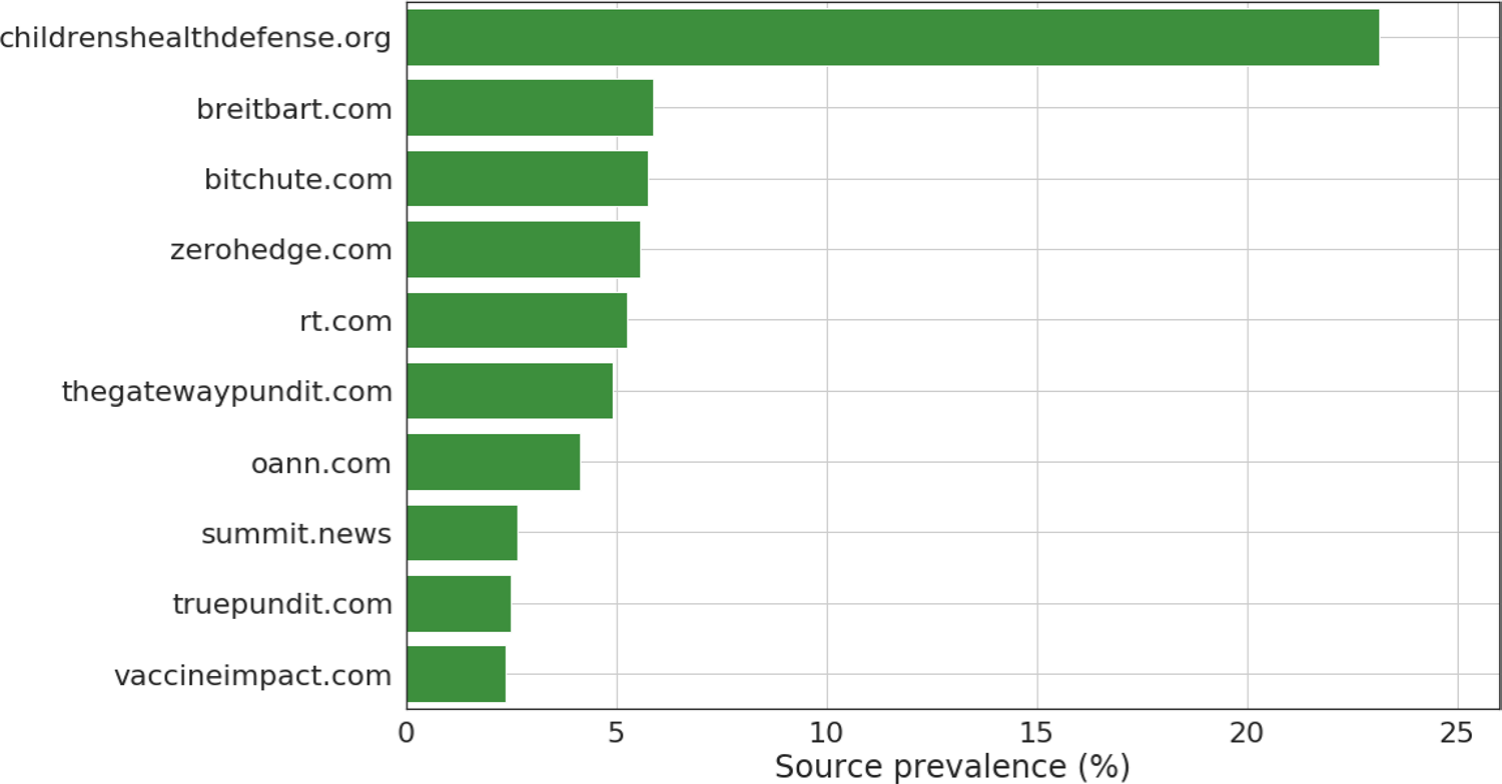
Top low-credibility sources. We considered tweets shared by users geolocated in the U.S. that link to a low-credibility source. Sources are ranked by percentage of the tweets considered.

Finally, Fig. 3 shows the most shared low-credibility sources. We note the large prevalence of one particular source, Children’s Health Defense, an anti-vaccination organization that has been identified as one of the main sources of misinformation on vaccines^34,35^. We did not observe significant differences in the top sources shared in Republican vs. Democratic majority states.

## Discussion

Our results provide evidence for the problem of geographical regions with lower levels of COVID-19 vaccine uptake, which may be driven by online misinformation. Considering variability across regions with low and high levels of misinformation, the best estimates from our data predict a ~ 20% decrease in vaccine uptake between states, and a ~ 67% increase in hesitancy rates across Democratic counties, across the full range of misinformation prevalence. At these levels of vaccine uptake, the data predict SARS-CoV-2 will remain endemic in many U.S. regions. This suggests a need to counter misinformation in order to promote vaccine uptake.

An important question is whether online misinformation drives vaccine hesitancy. Our analyses alone do not demonstrate a causal relationship between misinformation and vaccine refusal. Our work is at an ecological scale and vaccine-hesitant individuals are potentially more likely to post vaccine misinformation. However, at the individual level, a recent study^20^ found that exposure to online misinformation can increase vaccine hesitancy. Our work serves to provide evidence that those findings, which were obtained under controlled circumstances, scale to an ecological setting. Due to the fact that vaccine hesitancy and misinformation are socially reinforced, both ecological and individual relationships are important in demonstrating a causal link^36^. However, we are still unable to rule out confounding factors, so uncertainty remains about a causal link and further investigation is warranted.

Public opinion is very sensitive to the information ecosystem and sensational posts tend to spread widely and quickly^25^. Our results indicate that there is a geographical component to this spread, with opinions on vaccines spreading at a local scale. While social media users are not representative of the general public, existing evidence suggests that vaccine hesitancy flows across social networks^37^, providing a mechanism for the lateral spread of misinformation offline among those connected directly or indirectly to misinformation spreading online. More broadly, our results provide additional insight into the effects of information diffusion on human behavior and the spread of infectious diseases^38^.

A limitation of our findings is that we are not measuring the exposure, by geographical region, to misinformation on Twitter but rather the sharing activity of a subset of users. Besides, our analyses are based on data averaged over geographical regions. To account for group-level effects we present a number of sensitivity analyses, and note that our findings are consistent over two geographical scales. Our source-based approach to detect misinformation at scale might not capture the totality of misleading and harmful content related to vaccines, and many low-credibility sources publish a mixture of false and true information^39,40^. Our results are also limited to a small period of time. Finally, other factors might also influence vaccination hesitancy levels, including accessibility to vaccines, changes in COVID-19 infection and death rates, as well as legitimate reports about vaccine safety^41^.

Associations between online misinformation and detrimental offline effects, like the results presented here, call for better moderation of our information ecosystem. COVID-19 misinformation is shared overtly by known entities on major social media platforms^42^. While people have a constitutional right to free speech, it is important to maintain an environment where individuals have access to good information that benefits public health.

### Data and code availability

All measurements of vaccine uptake and vaccine hesitancy rates as well as socioeconomic, political, and demographic variables at the state and county level are publicly available in the online repository associated with this paper^43^. We also provide aggregated measurements of online misinformation shared by geolocated Twitter users. Results at the state and county level can be fully reproduced using the STATA scripts provided in the repository. Due to Twitter’s terms of use and service, we can only release IDs of the tweets present in our dataset, which can be reconstructed using the Twitter API. The IDs are accessible in the public dataset associated with the CoVaxxy project^17^ from the Observatory on Social Media at Indiana University.

## Supplementary Information

Supplementary Information.
