# Supplementary material for: Online misinformation is linked to early COVID-19 vaccination hesitancy and refusal
Source: Sci Rep. 2022 Apr 26;12:5966. doi: 10.1038/s41598-022-10070-w (PMC9043199; doi:10.1038/s41598-022-10070-w)
Supplement: Supplementary file 1 — Supplementary Information. [file 41598_2022_10070_MOESM1_ESM.docx]

# Supplemental Information for: **Online misinformation is linked to COVID-19 vaccination hesitancy and refusal**

Francesco Pierri, Brea L. Perry, Matthew R. DeVerna, Kai-Cheng Yang, Alessandro Flammini, Filippo Menczer and John Bryden

# Data collection and sources

## Twitter data

In our CoVaxxy[^1^](https://www.zotero.org/google-docs/?Kmve9N) project, we collected around 55 M English-language posts about vaccines on Twitter by means of the Twitter *POST statuses/filter v1.1 API*, in the period from January 4th, 2021 to March 25th, 2021. Data collection and analysis was done using the Extreme Science and Engineering Discovery Environment (XSEDE)[^2^](https://www.zotero.org/google-docs/?tmJ7g7).

To define as complete a set as possible of English language keywords related to vaccines, we employed a snowball sampling methodology in December 2020[^1^](https://www.zotero.org/google-docs/?J9hd2z) (see reference for full details on the data collection pipeline). The final list contains almost 80 keywords, and it is accessible in the online repository associated with the reference[^3^](https://www.zotero.org/google-docs/?B1eyiI). As a robustness test, we further perform sensitivity analyses using a restricted set of keywords (“vaccine”, “vaccinate”, “vaccination”, “vax”) which covers almost 95% of the total number of geolocated tweets. Results are equivalent to those presented in the main text and are described in the section “Sensitivity Analyses”.

To match Twitter posts with US states and counties, we first identified a collection of Twitter accounts that disclosed a location in their Twitter profile. We then employed the *carmen* Python library[^4^](https://www.zotero.org/google-docs/?N62nW4) to match each location to US states and counties. We were able to match around 1.67 *M* users to 50 US states, and a subset of 1.15 *M* users to over 1,300 US counties; the larger set accounts for a total number of almost 11 *M* shared tweets.

To analyze the spread of low-credibility information, we identified all URLs shared in Twitter posts that originated from a list of low-credibility sources, following a large corpus of literature[^5–9^](https://www.zotero.org/google-docs/?YRASR3). We employ the Iffy+ Misinfo/Disinfo list of low-credibility sources[^10^](https://www.zotero.org/google-docs/?TQIQhY), which is based on information provided by the Media Bias/Fact Check website (MBFC, <https://mediabiasfactcheck.com>), an independent organization that reviews and rates the reliability of news sources. As defined in the related methodology, political leaning is not a factor for inclusion. The list includes sites labeled by MBFC as having a “Very Low” or “Low” factual-reporting level as well as those classified as “Questionable” or “Conspiracy-Pseudoscience”. The list also includes fake-news websites flagged by BuzzFeed, FactCheck.org, PolitiFact, and Wikipedia, for a total number of 674 low-credibility sources.

Based on this list, we measure the prevalence of low-credibility information about vaccines in each region by (1) calculating the proportion of vaccine-related tweets containing URLs pointing to a low-credibility news website, for each geo-located account; and (2) taking the average of this proportion across all accounts within a specific region. We refer to this average as the state-wide (county-wide) prevalence of misinformation.

At the county level, we omit observations without vaccine hesitancy data (see next section), and we used different thresholds for the minimum number of geolocated accounts, respectively 10, 50, and 100. In the main paper, we present results when using 100 as a threshold. We provide sensitivity analyses using versions including counties with at least 10 and 50 Twitter accounts (see “Sensitivity Analyses” section). The larger threshold is likely to contain less error but also omits more counties.

## Election data

We use data provided by the MIT Election Lab to extract state-level returns for the 2020 US presidential election[^11^](https://www.zotero.org/google-docs/?DKQ4wd). For counties, we use data provided by Fox News, Politico, and the New York Times. They are publicly available at <https://github.com/tonmcg/US_County_Level_Election_Results_08-20>.

## Vaccine hesitancy data

To compute vaccine hesitancy rates in each state (county), we leverage daily COVID-19 Symptom Surveys produced by the Delphi Group at Carnegie Mellon University[^12^](https://www.zotero.org/google-docs/?v5xsMV). These surveys are voluntarily answered by a random sample of users on Facebook (total reported sample size *N* = 22,128,855). Within the Vaccination Indicators of the survey, we extract the estimated percentage of respondents (for each state/county) “who either have already received a COVID vaccine or would definitely or probably choose to get vaccinated, if a vaccine were offered to them today.” Results are available daily, for all 50 US states and for 764 US counties. We compute state-wide (county-wide) vaccine hesitancy rates by taking the proportion of negative responses in the period from January 4th to March 25th.

## Vaccine uptake data

Vaccination uptake statistics are derived from the Centers for Disease Control and Prevention (CDC) dataset (<https://covid.cdc.gov/covid-data-tracker/#vaccinations>). Doses monitored for each state include those administered in jurisdictional partner clinics, retail pharmacies, long-term care facilities, Federal Emergency Management Agency partner sites, Health Resources and Services Administration partner sites, and federal facilities. The data have been compiled on a daily basis by *ourworldindata.org*, and we have downloaded them for the period from January 12 to March 25, 2021. The data are available at <https://github.com/owid/covid-19-data/tree/master/public/data/vaccinations>.

## COVID-19 data

We extracted the number of COVID-19 cases and fatalities at the state and county level based on reports made by USAFacts (<https://usafacts.org>). In particular, we summed the number of daily confirmed COVID-19 cases and fatalities, referring to these as “recent”, in the period from January 4 to March 25, 2021. We then computed the cumulative number of cases and fatalities on March 25th, referring to these as “total”.

## Socioeconomic data

To include socioeconomic covariates in our regression model, we use data from the Atlas of Rural and Small-Town America (available at <https://www.ers.usda.gov/data-products/atlas-of-rural-and-small-town-america/>), which includes data at the state and county level from the American Community Survey (ACS), the Bureau of Labor Statistics, and other sources. We employ data last updated on July 2, 2020, which include county population estimates and annual unemployment/employment data for 2019.

County-level measurements about religion are derived from surveys by the Association of Religion Data Archives (accessible at <https://www.thearda.com/Archive/ChCounty.asp>).

# Additional correlation results

Figures S1 and S2 present additional results about correlations between vaccine demand, vaccine hesitancy, political partisanship, and online misinformation at state and county levels.

# Main findings from regression analysis

Table S1 presents results from the weighted (Models 1 and 2) and ordinary (Models 3 and 4) least-squares regression of state-level vaccine hesitancy and vaccination rate, respectively, on covariates. As shown in Model 1, the misinformation variable and the percent of GOP voters explain nearly 80% of the variation in vaccine hesitancy at the state level. These predictors remain significant after the addition of multiple control variables (see Model 2). Misinformation and republican vote percentage explain nearly half of the variation in vaccination rate (see Model 3), and are also significantly associated with vaccination rate at the state level net of controls (see Model 4).


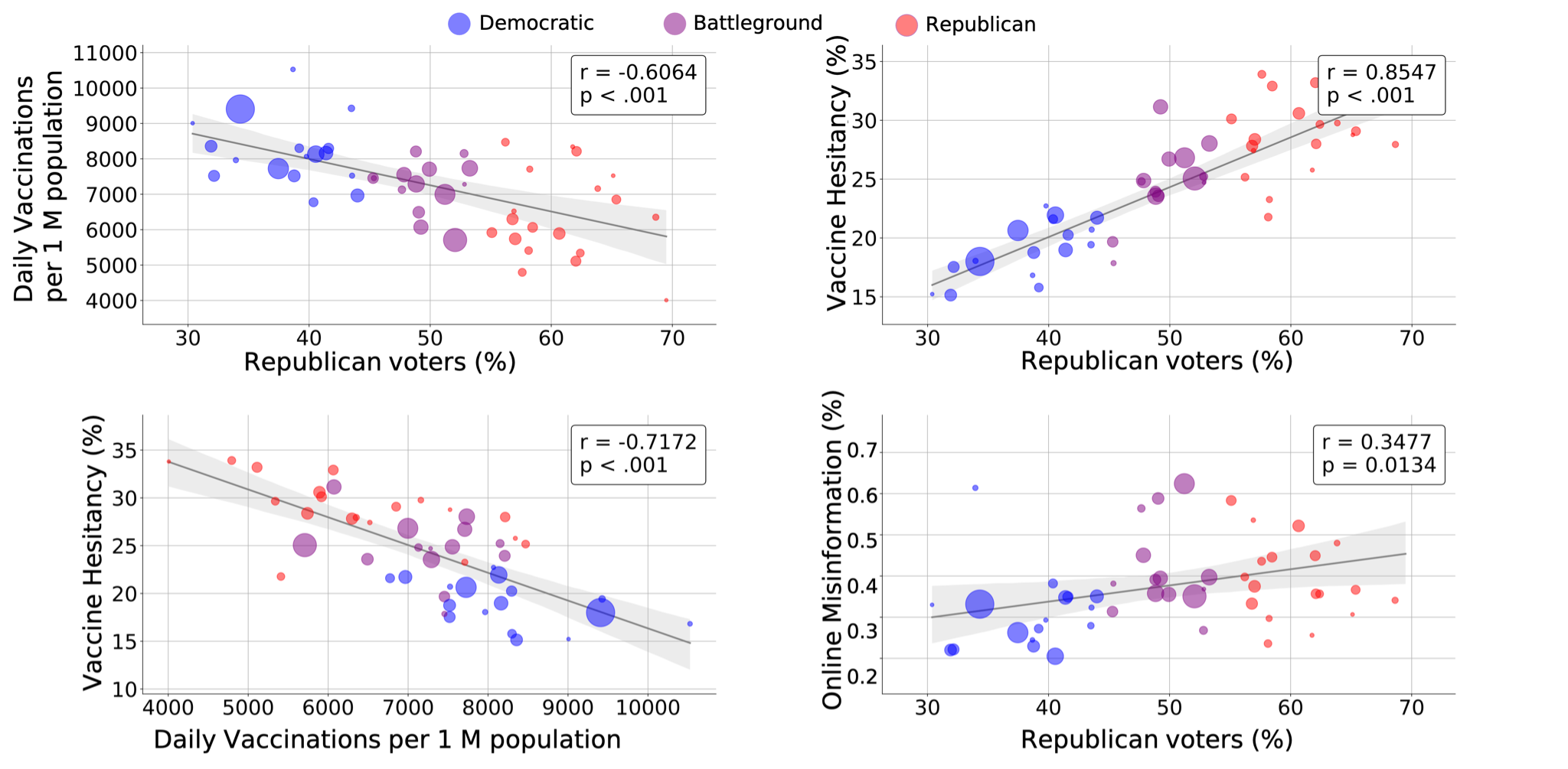


**Figure S1. Correlations between vaccine demand, vaccine hesitancy, political partisanship, and online misinformation at the state level.** Vaccine demand is computed as the mean number of daily vaccinations per million population in the period 19-25 March 2021. Vaccine hesitancy corresponds to the proportion of individuals who would not get vaccinated according to Facebook daily surveys administered in the period from January 4th to March 25th, 2021. Partisanship is measured as the percentage of Republican voters in the 2020 US Presidential elections. Online misinformation about vaccines shared on Twitter is measured during the period from Jan 4th to March 25th, 2021. Each dot represents a U.S. state, sized according to population and colored according to Republican vote share (battleground states have a share between 45% and 55%).


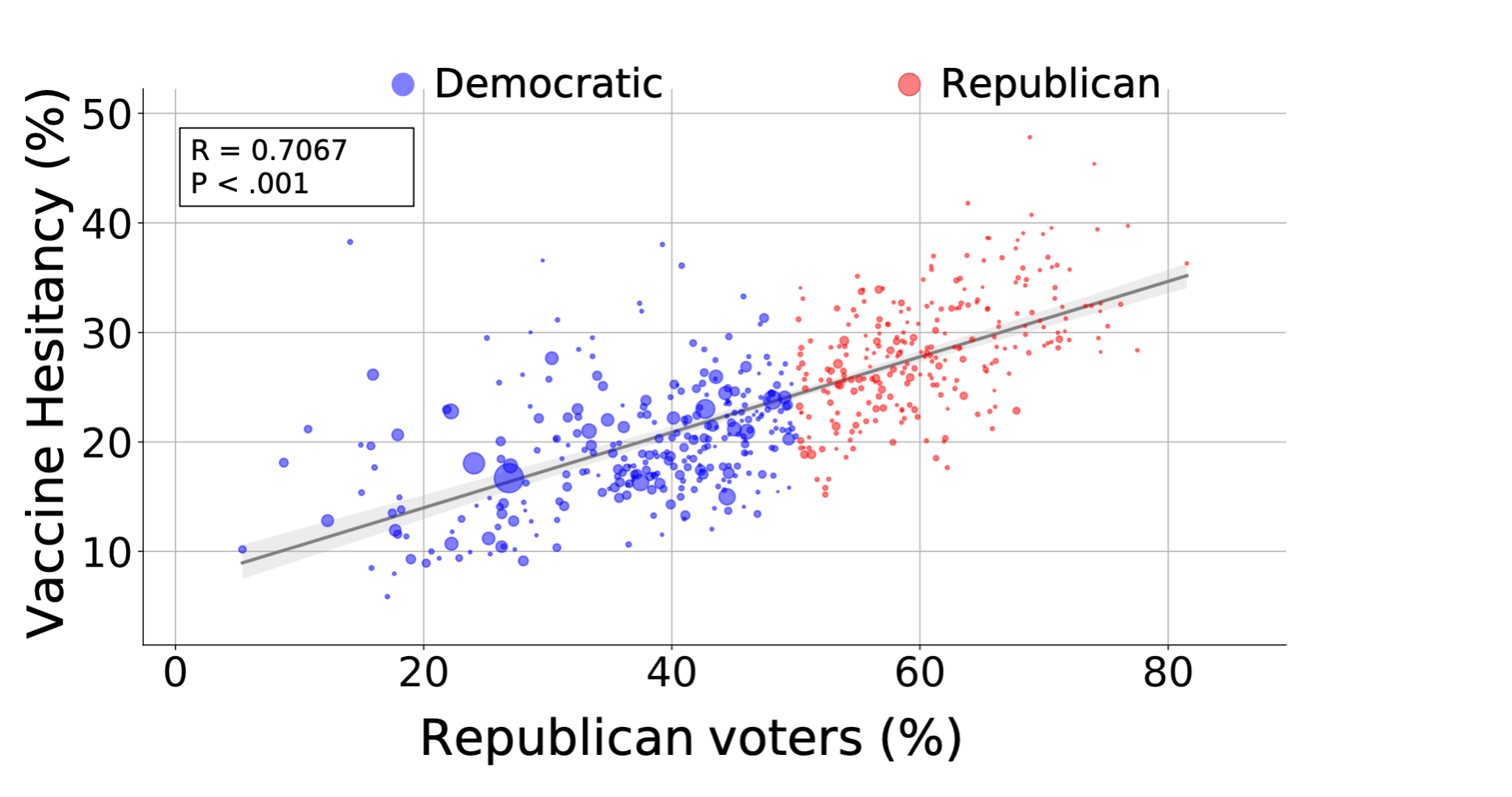


**Figure S2. Political partisanship is correlated with vaccine hesitancy at the U.S. county level.** Vaccine hesitancy corresponds to the proportion of individuals who would not get vaccinated according to Facebook daily surveys administered in the period from January 4th to March 25th, 2021. Partisanship is measured as the percentage of Republican voters in the 2020 US Presidential elections. Each dot represents a U.S. county, sized according to population and colored according to Republican vote share.

# Sensitivity analyses

We conduct a set of sensitivity analyses to ensure that our findings are robust to alternative variable and model specifications. First, we run standard diagnostics for nonlinearity, skewness, multicollinearity, and heteroskedasticity, correcting any problems we discover. Second, because the misinformation measure at the state level is slightly positively skewed, we conduct a model using a natural logarithmic transformation of mean percent misinformation. Results from these models are consistent with the main findings (Table S2). The untransformed variable has a better model fit (lower BIC). Third, because the effect of misinformation may depend on political partisanship, we test for an interaction between misinformation and the percent of GOP voters. There is no evidence of such interaction at the state level. Fourth, we rerun the above models using versions of the mean percentage of vaccine-related misinformation shared by Twitter users by considering a restricted set of keywords to gather tweets (see previous “Twitter Data” section). As shown in Table S3, findings are consistent and robust to this alternate definition of misinformation sharing.

We also conduct a similar set of sensitivity analyses at the county level. First, we test multiple versions of the misinformation variable, which is highly skewed and zero-inflated at the county level. We use the log-transformed version for the main findings due to the best model fit, but obtain significant results with the untransformed variable and very similar findings with a polynomial model that also captures the nonlinear relationship between misinformation and vaccine hesitancy. Second, we test for an interaction between misinformation and percent of GOP voters, finding that being in a majority Republican versus Democratic state moderates the association between misinformation and vaccine hesitancy (Table S4). A scatterplot of republican and democratic-leaning counties confirms the moderation finding (Fig.2 in the main manuscript). Third, we run models adding the number of tweets per county as a control variable to address variation in the volume of Twitter activity across counties. Adding this covariate did not affect results. Fourth, as at the state level, we generate versions of the vaccine misinformation variable using a restricted set of keywords. Again, these results are consistent with our main findings (Table S5). Fifth, we examine the robustness of the threshold of 100 Twitter accounts per county for inclusion in the analysis, setting thresholds of 50 and 10. These results are similar to the main findings (Tables S6 and S7), demonstrating that results are robust to different variable specifications.

To confirm the relationship between misinformation and GOP vote share, we compute a negative binomial regression model predicting mean percent information (untransformed) at the county level using percent GOP vote and a set of control variables. This multivariate analysis confirms the bivariate correlation, indicating a strong relationship between these factors net of potential confounding variables (Table S8).

Table S9 describes all the covariates considered in the regression analyses. Table S10 and S11 provide results of the OLS regression for the Granger causality analysis respectively at county and state level.

Table S1. Weighted/ordinary least squares regression of state-level percent vaccine hesitancy

and daily vaccination rate per million on misinformation and covariates (N=50 states). In this and the following tables, columns correspond to different models.

|  | (1) | (2) | (3) | (4) |
| --- | --- | --- | --- | --- |
|  | Vaccine hesitancy | Vaccine hesitancy | Vaccination rate | Vaccination rate |
|  | b (SE) | b (SE) | b (SE) | b (SE) |
| Mean % low credibility tweets | 8.093^*^ | 6.877^**^ | -3444.858^**^ | -3518.002^**^ |
|  | (3.04) | (2.43) | (1240.20) | (1277.08) |
| % GOP vote (10% change) | 3.996^***^ | 2.960^***^ | -606.567^***^ | -640.319^**^ |
|  | (0.38) | (0.42) | (140.32) | (208.11) |
| % below poverty line |  | 0.530^**^ |  | 18.173 |
|  |  | (0.15) |  | (81.84) |
| % aged 65+ |  | -0.197 |  | 171.533 |
|  |  | (0.15) |  | (100.14) |
| % Asian |  | 0.011 |  | 13.213 |
|  |  | (0.07) |  | (27.74) |
| % Black |  | 0.124^**^ |  | -40.491 |
|  |  | (0.04) |  | (22.54) |
| % Hispanic |  | -0.066^*^ |  | 4.564 |
|  |  | (0.03) |  | (19.71) |
| % Indigenous |  | -0.138 |  | 71.890 |
|  |  | (0.12) |  | (51.00) |
| COVID deaths/thousand |  | -0.221 |  | 217.490 |
|  |  | (0.42) |  | (262.06) |
| Constant | 1.858 | 3.024 | 11586.785^***^ | 9126.137^***^ |
|  | (1.65) | (2.72) | (708.20) | (1537.38) |
| *R^2^* | 0.797^***^ | 0.937^***^ | 0.457^***^ | 0.641^***^ |
| *BIC* | 225.217 | 194.454 | 836.580 | 843.252 |

Notes: Vaccine hesitancy is based on state-level means from Facebook survey data. The vaccination rate is vaccines administered per million (CDC data). For models predicting vaccine hesitancy (i.e., state means), analytic weights based on sample size are applied. Unstandardized betas and standard errors are provided. ^*^ *p* < 0.05, ^**^ *p* < 0.01, ^***^ *p* < 0.001

Table S2. Weighted/ordinary least squares regression of state-level percent vaccine hesitancy and daily vaccination rate per million on misinformation (logged) and covariates (N=50 states).

|  | (1) | (2) | (3) | (4) |
| --- | --- | --- | --- | --- |
|  | Vaccine hesitancy | Vaccine hesitancy | Vaccination rate | Vaccination rate |
|  | b (SE) | b (SE) | b (SE) | b (SE) |
| Logged mean % low cred tweets | 4.136^**^ | 3.257^**^ | -1669.206^*^ | -1593.010^*^ |
|  | (1.53) | (1.19) | (636.52) | (660.59) |
| % GOP vote (10% change) | 3.945^***^ | 2.962^***^ | -601.418^***^ | -676.915^**^ |
|  | (0.38) | (0.42) | (143.03) | (210.70) |
| % below poverty line |  | 0.515^**^ |  | 29.711 |
|  |  | (0.15) |  | (83.31) |
| % aged 65+ |  | -0.158 |  | 158.518 |
|  |  | (0.14) |  | (101.53) |
| % Asian |  | 0.009 |  | 8.878 |
|  |  | (0.07) |  | (28.09) |
| % Black |  | 0.130^**^ |  | -42.750 |
|  |  | (0.04) |  | (22.90) |
| % Hispanic |  | -0.062^*^ |  | 1.398 |
|  |  | (0.03) |  | (19.93) |
| % Indigenous |  | -0.129 |  | 70.503 |
|  |  | (0.12) |  | (51.98) |
| COVID deaths/thousand |  | -0.235 |  | 224.368 |
|  |  | (0.42) |  | (268.26) |
| Constant | 8.318^**^ | 7.683 | 8981.085^***^ | 6852.773^**^ |
|  | (2.63) | (3.90) | (1015.40) | (2048.22) |
| *R^2^* | 0.798^***^ | 0.936^***^ | 0.448^***^ | 0.627^***^ |
| *BIC* | 225.049 | 194.982 | 837.352 | 845.150 |

Notes: Vaccine hesitancy is based on state-level means from Facebook survey data. The vaccination rate is actual vaccines administered per million (CDC data). For models predicting vaccine hesitancy (i.e., state means), analytic weights based on sample size are applied. Unstandardized betas and standard errors are provided. ^*^ *p* < 0.05, ^**^ *p* < 0.01, ^***^ *p* < 0.001

Table S3. Weighted/ordinary least squares regression of state-level percent vaccine hesitancy and daily vaccination rate per million on misinformation (restricted key words) and covariates (N=50 states).

|  | (1) | (2) | (3) | (4) |
| --- | --- | --- | --- | --- |
|  | Vaccine hesitancy | Vaccine hesitancy | Vaccination rate | Vaccination rate |
|  | b (SE) | b (SE) | b (SE) | b (SE) |
| Mean % low credibility tweets | 8.320^**^ | 7.108^**^ | -3342.575^**^ | -3517.510^**^ |
|  | (2.97) | (2.37) | (1200.22) | (1236.41) |
| % GOP vote (10% change) | 3.982^***^ | 2.944^***^ | -611.854^***^ | -648.565^**^ |
|  | (0.37) | (0.41) | (139.58) | (204.44) |
| % below poverty line |  | 0.517^**^ |  | 27.129 |
|  |  | (0.15) |  | (81.32) |
| % aged 65+ |  | -0.206 |  | 170.945 |
|  |  | (0.15) |  | (99.35) |
| % Asian |  | 0.003 |  | 16.019 |
|  |  | (0.07) |  | (27.87) |
| % Black |  | 0.125^**^ |  | -42.464 |
|  |  | (0.04) |  | (22.25) |
| % Hispanic |  | -0.065^*^ |  | 2.774 |
|  |  | (0.03) |  | (19.42) |
| % Indigenous |  | -0.132 |  | 68.678 |
|  |  | (0.12) |  | (50.75) |
| COVID deaths/thousand |  | -0.216 |  | 225.119 |
|  |  | (0.42) |  | (259.70) |
| Constant | 1.841 | 3.313 | 11575.126^***^ | 9085.430^***^ |
|  | (1.64) | (2.71) | (706.47) | (1530.36) |
| *R^2^* | 0.800^***^ | 0.938^***^ | 0.457^***^ | 0.645^***^ |
| *BIC* | 224.530 | 193.465 | 836.543 | 842.724 |

Notes: Vaccine hesitancy is based on state-level means from Facebook survey data. The vaccination rate is actual vaccines administered per million (CDC data). For models predicting vaccine hesitancy (i.e., state means), analytic weights based on sample size are applied. Unstandardized betas and standard errors are provided. ^*^ *p* < 0.05, ^**^ *p* < 0.01, ^***^ *p* < 0.001

Table S4. Weighted least squares regression of county-level percent vaccine hesitancy on misinformation (logged) and covariates (N=548 counties, minimum 100 accounts/county).

|  | (1) | (2) | (3) | (4) |
| --- | --- | --- | --- | --- |
|  | b (SE) | b (SE) | b (SE) | b (SE) |
| Logged mean % low credibility tweets | 1.411^**^ | 4.304^***^ | 1.018^***^ | 4.278^***^ |
|  | (0.47) | (0.78) | (0.28) | (0.59) |
| % GOP vote (10% change) | 2.926^***^ |  | 3.663^***^ |  |
|  | (0.29) |  | (0.16) |  |
| Majority GOP state (1=GOP; 0=Dem) |  | 3.892^***^ |  | 3.340^***^ |
|  |  | (1.02) |  | (0.66) |
| GOP state * Logged low credibility |  | -3.585^***^ |  | -3.414^***^ |
|  |  | (0.99) |  | (0.76) |
| % below poverty line |  |  | 0.376^***^ | 0.398^***^ |
|  |  |  | (0.07) | (0.08) |
| % aged 65+ |  |  | -0.056 | -0.091 |
|  |  |  | (0.05) | (0.05) |
| % Asian |  |  | 0.028 | -0.173^**^ |
|  |  |  | (0.03) | (0.05) |
| % Black |  |  | 0.202^***^ | 0.090^***^ |
|  |  |  | (0.02) | (0.03) |
| % Hispanic |  |  | 0.002 | -0.030 |
|  |  |  | (0.02) | (0.02) |
| % Indigenous |  |  | 0.033 | -0.108 |
|  |  |  | (0.19) | (0.14) |
| Rural-urban continuum code |  |  | 0.447 | 0.617 |
|  |  |  | (0.26) | (0.34) |
| COVID deaths/thousand |  |  | 0.547^*^ | 0.925^**^ |
|  |  |  | (0.27) | (0.29) |
| Constant | 10.227^***^ | 23.668^***^ | -1.535 | 17.834^***^ |
|  | (1.63) | (1.03) | (1.12) | (1.45) |
| *R^2^* | 0.500^***^ | 0.419^***^ | 0.805^***^ | 0.662^***^ |
| *BIC* | 3151.490 | 3240.010 | 2686.806 | 2993.820 |

Notes: Vaccine hesitancy is based on county-level means from Facebook survey data. Misinformation is measured using mean percent of low credibility tweets for counties with at least 100 Twitter accounts. Analytic weights based on Facebook survey sample size are applied, and models use cluster robust standard errors to account for counties being nested in states. Unstandardized betas and standard errors are provided. ^*^ *p* < 0.05, ^**^ *p* < 0.01, ^***^ *p* < 0.001

Table S5. Weighted least squares regression of county-level percent vaccine hesitancy on misinformation (logged, restricted key words) and covariates (N=548 counties, minimum 100 accounts/county).

|  | (1) | (2) | (3) | (4) |
| --- | --- | --- | --- | --- |
|  | b (SE) | b (SE) | b (SE) | b (SE) |
| Logged mean % low credibility tweets | 1.510^**^ | 4.382^***^ | 1.074^***^ | 4.319^***^ |
|  | (0.46) | (0.73) | (0.27) | (0.53) |
| % GOP vote (10% change) | 2.905^***^ |  | 3.641^***^ |  |
|  | (0.29) |  | (0.15) |  |
| Majority GOP state (1=GOP; 0=Dem) |  | 12.010^***^ |  | 11.132^***^ |
|  |  | (1.49) |  | (1.16) |
| GOP state * Logged low credibility |  | -3.530^***^ |  | -3.392^***^ |
|  |  | (0.94) |  | (0.70) |
| % below poverty line |  |  | 0.375^***^ | 0.394^***^ |
|  |  |  | (0.07) | (0.08) |
| % aged 65+ |  |  | -0.058 | -0.095 |
|  |  |  | (0.05) | (0.05) |
| % Asian |  |  | 0.028 | -0.171^**^ |
|  |  |  | (0.03) | (0.05) |
| % Black |  |  | 0.202^***^ | 0.091^***^ |
|  |  |  | (0.02) | (0.03) |
| % Hispanic |  |  | 0.002 | -0.030 |
|  |  |  | (0.02) | (0.02) |
| % Indigenous |  |  | 0.038 | -0.101 |
|  |  |  | (0.19) | (0.13) |
| Rural-urban continuum code |  |  | 0.451 | 0.648 |
|  |  |  | (0.26) | (0.33) |
| COVID deaths/thousand |  |  | 0.546^*^ | 0.916^**^ |
|  |  |  | (0.26) | (0.28) |
| Constant | 6.937^***^ | 13.673^***^ | -3.849^***^ | 7.981^***^ |
|  | (1.14) | (0.95) | (0.93) | (1.29) |
| *R^2^* | 0.501^***^ | 0.423^***^ | 0.805^***^ | 0.665^***^ |
| *BIC* | 3136.899 | 3222.391 | 2673.021 | 2975.819 |

Notes: Vaccine hesitancy is based on county-level means from Facebook survey data. Misinformation is measured using mean percent of low credibility tweets for counties with at least 100 Twitter accounts. Analytic weights based on Facebook survey sample size are applied, and models use cluster robust standard errors to account for counties being nested in states. Unstandardized betas and standard errors are provided. ^*^ *p* < 0.05, ^**^ *p* < 0.01, ^***^ *p* < 0.001

Table S6. Weighted least squares regression of county-level percent vaccine hesitancy on misinformation (logged) and covariates (N=658 counties, minimum 10 accounts/county).

|  | (1) | (2) | (3) | (4) |
| --- | --- | --- | --- | --- |
|  | b (SE) | b (SE) | b (SE) | b (SE) |
| Logged mean % low credibility tweets | 1.078^*^ | 3.252^**^ | 0.941^***^ | 3.673^***^ |
|  | (0.47) | (1.11) | (0.22) | (0.75) |
| % GOP vote (10% change) | 3.140^***^ |  | 3.748^***^ |  |
|  | (0.29) |  | (0.15) |  |
| Majority GOP state (1=GOP; 0=Dem) |  | 5.627^***^ |  | 4.247^***^ |
|  |  | (1.55) |  | (0.85) |
| GOP state * Logged low credibility |  | -2.467^*^ |  | -2.746^**^ |
|  |  | (1.16) |  | (0.84) |
| % below poverty line |  |  | 0.369^***^ | 0.378^***^ |
|  |  |  | (0.07) | (0.07) |
| % aged 65+ |  |  | -0.059 | -0.114^*^ |
|  |  |  | (0.06) | (0.05) |
| % Asian |  |  | 0.023 | -0.223^***^ |
|  |  |  | (0.02) | (0.05) |
| % Black |  |  | 0.204^***^ | 0.089^***^ |
|  |  |  | (0.02) | (0.02) |
| % Hispanic |  |  | 0.002 | -0.030 |
|  |  |  | (0.02) | (0.02) |
| % Indigenous |  |  | -0.002 | -0.065 |
|  |  |  | (0.12) | (0.11) |
| Rural-urban continuum code |  |  | 0.600^**^ | 0.749^*^ |
|  |  |  | (0.22) | (0.32) |
| COVID deaths/thousand |  |  | 0.549^*^ | 1.054^***^ |
|  |  |  | (0.27) | (0.29) |
| Constant | 9.047^***^ | 22.464^***^ | -2.034 | 17.582^***^ |
|  | (1.65) | (1.58) | (1.07) | (1.56) |
| *R^2^* | 0.534^***^ | 0.421^***^ | 0.812^***^ | 0.664^***^ |
| *BIC* | 3796.413 | 3945.657 | 3251.830 | 3639.761 |

Notes: Vaccine hesitancy is based on county-level means from Facebook survey data. Misinformation is measured using mean percent of low credibility tweets for counties with at least 10 Twitter accounts. Analytic weights based on Facebook survey sample size are applied, and models use cluster robust standard errors to account for counties being nested in states. Unstandardized betas and standard errors are provided. ^*^ *p* < 0.05, ^**^ *p* < 0.01, ^***^ *p* < 0.001

Table S7. Weighted least squares regression of county-level percent vaccine hesitancy on misinformation (logged) and covariates (N=628 counties, minimum 50 accounts/county).

|  | (1) | (2) | (3) | (4) |
| --- | --- | --- | --- | --- |
|  | b (SE) | b (SE) | b (SE) | b (SE) |
| Logged mean % low credibility tweets | 1.347^**^ | 4.241^***^ | 1.028^***^ | 4.233^***^ |
|  | (0.42) | (0.78) | (0.24) | (0.59) |
| % GOP vote (10% change) | 3.039^***^ |  | 3.718^***^ |  |
|  | (0.27) |  | (0.15) |  |
| Majority GOP state (1=GOP; 0=Dem) |  | 4.480^***^ |  | 3.731^***^ |
|  |  | (0.99) |  | (0.65) |
| GOP state * Logged low credibility |  | -3.350^***^ |  | -3.236^***^ |
|  |  | (0.90) |  | (0.69) |
| % below poverty line |  |  | 0.378^***^ | 0.407^***^ |
|  |  |  | (0.07) | (0.08) |
| % aged 65+ |  |  | -0.059 | -0.102 |
|  |  |  | (0.06) | (0.05) |
| % Asian |  |  | 0.030 | -0.173^**^ |
|  |  |  | (0.03) | (0.05) |
| % Black |  |  | 0.202^***^ | 0.087^***^ |
|  |  |  | (0.02) | (0.02) |
| % Hispanic |  |  | 0.001 | -0.034 |
|  |  |  | (0.02) | (0.02) |
| % Indigenous |  |  | -0.008 | -0.083 |
|  |  |  | (0.12) | (0.10) |
| Rural-urban continuum code |  |  | 0.559^*^ | 0.716^*^ |
|  |  |  | (0.23) | (0.31) |
| COVID deaths/thousand |  |  | 0.538 | 0.972^**^ |
|  |  |  | (0.27) | (0.28) |
| Constant | 9.757^***^ | 23.600^***^ | -1.842 | 17.708^***^ |
|  | (1.48) | (1.03) | (1.09) | (1.49) |
| *R^2^* | 0.524^***^ | 0.439^***^ | 0.809^***^ | 0.667^***^ |
| *BIC* | 3619.976 | 3729.469 | 3099.337 | 3453.070 |

Notes: Vaccine hesitancy is based on county-level means from Facebook survey data. Misinformation is measured using mean percent of low credibility tweets for counties with at least 50 Twitter accounts. Analytic weights based on Facebook survey sample size are applied, and models use cluster robust standard errors to account for counties being nested in states. Unstandardized betas and standard errors are provided. ^*^ *p* < 0.05, ^**^ *p* < 0.01, ^***^ *p* < 0.001

Table S8. Negative binomial regression of

county-level misinformation on

percent GOP vote and covariates (N=548

counties).

|  | b (SE) |
| --- | --- |
| % GOP vote (10% change) | 0.263^***^ |
|  | (0.04) |
| % below poverty line | -0.019^*^ |
|  | (0.01) |
| % aged 65+ | 0.043^***^ |
|  | (0.01) |
| % Asian | 0.017 |
|  | (0.01) |
| % Black | 0.013^***^ |
|  | (0.00) |
| % Hispanic | 0.006^*^ |
|  | (0.00) |
| % Indigenous | 0.031^*^ |
|  | (0.02) |
| Rural-urban continuum code | -0.068 |
|  | (0.04) |
| COVID deaths/thousand | -0.098 |
|  | (0.06) |
| Constant | -2.647^***^ |
|  | (0.23) |
| *Wald chi-squared* | 232.330^***^ |
| *BIC* | 774.836 |

Notes: Misinformation is measured using mean percent

of low credibility tweets for counties with at least 100

Twitter accounts. Models use cluster robust standard

errors to account for counties being nested in states.

Negative binomial regression is employed due to zero-

inflated Poisson distribution. Unstandardized betas and

standard errors are provided. ^*^ *p* < 0.05, ^**^ *p* < 0.01,

^***^ *p* < 0.001

Table S9. Description of covariates used during analyses.

|  |  |  |  |
| --- | --- | --- | --- |
| Stata variable | Description | Year | Source |
| vaxrate | Daily number of people vaccinated per million | 2021 | Centers for Disease Control and Prevention |
| lowcred | Mean percentage of low credibility shared  (per user) | 2021 | Twitter API |
| loglowcred | Natural logarithm of the mean percentage of low credibility shared  (per user) | 2021 | Twitter API |
| propgop | Proportion of votes for Republican candidate | 2020 | Fox News, Politico, New York Times |
| covidmortality | Total COVID 19 deaths | 2021 | Centers for Disease Control and Prevention |
| population | Census Population | 2010 | United States Census |
| vMedHHInc | Median Household Income | 2010 | United States Department of Agriculture  (Atlas of Rural and Small-Town America) |
| ppoverty | Percentage of people of all ages in poverty | 2019 | United States Department of Agriculture  (County-Level Datasets) |
| vPercBachelors | Percent of adults with a bachelor's degree or higher | 2015-2019 | United States Department of Agriculture  (County-Level Datasets) |
| vUnemployment_rate_2019 | Unemployment rate | 2019 | United States Department of Agriculture  (County-Level Datasets) |
| vTOTRATE | Rates of religious adherence per 1,000 population  (200+ religions) | 2010 | Association of Religious Data Archives |
| vUnder18Pct2010 | Percentage of population age 18 years or younger | 2010 | United States Department of Agriculture  (Atlas of Rural and Small-Town America) |
| vAge65AndOlderPct2010 | Percentage of population age 65 years or older | 2010 | United States Department of Agriculture  (Atlas of Rural and Small-Town America) |
| vAsianNonHispPct2010 | Percentage of population Asians  (Non-Hispanic) | 2010 | United States Department of Agriculture  (Atlas of Rural and Small-Town America) |
| vBlackNonHispPct2010 | Percentage of population Black  (Non-Hispanic) | 2010 | United States Department of Agriculture  (Atlas of Rural and Small-Town America) |
| vHispanicPct2010 | Percentage of population Hispanic | 2010 | United States Department of Agriculture  (Atlas of Rural and Small-Town America) |
| vNatAmNonHispPct2010 | Percentage of population Native American  (Non-Hispanic) | 2010 | United States Department of Agriculture  (Atlas of Rural and Small-Town America) |

# Table S10. Ordinary Least Squares regression of lagged variates for Granger Causality analysis. (N = 610 counties).

|  | (1) | (2) | (3) | (4) | (5) | (6) |
| --- | --- | --- | --- | --- | --- | --- |
|  | coef | std err | t | P>\|t\| | [0.025 | 0.975] |
| hesitancy t-1 | 0.8852 | 0.005 | 174.943 | 0 | 0.875 | 0.895 |
| hesitancy t-2 | 0.0039 | 0.007 | 0.571 | 0.568 | -0.009 | 0.017 |
| hesitancy t-3 | -0.0044 | 0.007 | -0.645 | 0.519 | -0.018 | 0.009 |
| hesitancy t-4 | -0.0004 | 0.007 | -0.061 | 0.951 | -0.014 | 0.013 |
| hesitancy t-5 | 0.0074 | 0.007 | 1.088 | 0.277 | -0.006 | 0.021 |
| hesitancy t-6 | -0.124 | 0.005 | -24.543 | 0 | -0.134 | -0.114 |
| misinfo t-1 | 0.006 | 0.004 | 1.362 | 0.173 | -0.003 | 0.015 |
| misinfo t-2 | 0.0087 | 0.004 | 1.972 | 0.049 | 5.36E-05 | 0.017 |
| misinfo t-3 | 0.0156 | 0.004 | 3.598 | 0 | 0.007 | 0.024 |
| misinfo t-4 | 0.0027 | 0.004 | 0.625 | 0.532 | -0.006 | 0.011 |
| misinfo t-5 | -0.0014 | 0.004 | -0.337 | 0.736 | -0.01 | 0.007 |
| misinfo t-6 | 0.0179 | 0.004 | 4.396 | 0 | 0.01 | 0.026 |
| AIC: | 56910 |  |  |  |  |  |
| R-squared (uncentered): | 0.743 |  |  |  |  |  |
|  |  |  |  |  |  |  |
| Null model |  |  |  |  |  |  |
|  | (1) | (2) | (3) | (4) | (5) | (6) |
|  | coef | std err | t | P>\|t\| | [0.025 | 0.975] |
| hesitancy t-1 | 0.8854 | 0.005 | 174.954 | 0 | 0.875 | 0.895 |
| hesitancy t-2 | 0.0037 | 0.007 | 0.549 | 0.583 | -0.01 | 0.017 |
| hesitancy t-3 | -0.0041 | 0.007 | -0.605 | 0.545 | -0.017 | 0.009 |
| hesitancy t-4 | -0.0005 | 0.007 | -0.079 | 0.937 | -0.014 | 0.013 |
| hesitancy t-5 | 0.0076 | 0.007 | 1.128 | 0.26 | -0.006 | 0.021 |
| hesitancy t-6 | -0.1239 | 0.005 | -24.526 | 0 | -0.134 | -0.114 |
| R-squared (uncentered): | 0.743 |  |  |  |  |  |
| AIC: | 56940 |  |  |  |  |  |

#

# Table S11. Ordinary Least Squares regression of lagged variates for Granger Causality analysis. (N = 50 states).

|  | (1) | (2) | (3) | (4) | (5) | (6) |
| --- | --- | --- | --- | --- | --- | --- |
|  | coef | std err | t | P>\|t\| | [0.025 | 0.975] |
| hesitancy t-1 | 0.9599 | 0.016 | 58.889 | 0 | 0.928 | 0.992 |
| hesitancy t-2 | 0.024 | 0.023 | 1.062 | 0.288 | -0.02 | 0.068 |
| hesitancy t-3 | -0.0748 | 0.022 | -3.325 | 0.001 | -0.119 | -0.031 |
| hesitancy t-4 | 0.1014 | 0.023 | 4.501 | 0 | 0.057 | 0.146 |
| hesitancy t-5 | -0.0904 | 0.023 | -3.988 | 0 | -0.135 | -0.046 |
| hesitancy t-6 | -0.0533 | 0.016 | -3.268 | 0.001 | -0.085 | -0.021 |
| misinfo t-1 | 0.0016 | 0.006 | 0.262 | 0.793 | -0.011 | 0.014 |
| misinfo t-2 | 0.021 | 0.006 | 3.351 | 0.001 | 0.009 | 0.033 |
| misinfo t-3 | 0.0018 | 0.006 | 0.295 | 0.768 | -0.01 | 0.014 |
| misinfo t-4 | -0.0161 | 0.006 | -2.603 | 0.009 | -0.028 | -0.004 |
| misinfo t-5 | 0.0133 | 0.006 | 2.153 | 0.031 | 0.001 | 0.025 |
| misinfo t-6 | 0.0003 | 0.006 | 0.044 | 0.965 | -0.012 | 0.012 |
| R-squared (uncentered): | 0.842 |  |  |  |  |  |
| AIC: | 3133 |  |  |  |  |  |
|  |  |  |  |  |  |  |
| Null model |  |  |  |  |  |  |
|  | (1) | (2) | (3) | (4) | (5) | (6) |
|  | coef | std err | t | P>\|t\| | [0.025 | 0.975] |
| hesitancy t-1 | 0.9593 | 0.016 | 58.935 | 0 | 0.927 | 0.991 |
| hesitancy t-2 | 0.0254 | 0.023 | 1.127 | 0.26 | -0.019 | 0.07 |
| hesitancy t-3 | -0.0725 | 0.023 | -3.22 | 0.001 | -0.117 | -0.028 |
| hesitancy t-4 | 0.0982 | 0.023 | 4.353 | 0 | 0.054 | 0.142 |
| hesitancy t-5 | -0.0879 | 0.023 | -3.873 | 0 | -0.132 | -0.043 |
| hesitancy t-6 | -0.0548 | 0.016 | -3.358 | 0.001 | -0.087 | -0.023 |
| R-squared (uncentered): | 0.841 |  |  |  |  |  |
| AIC: | 3143 |  |  |  |  |  |

#

# Supplementary Bibliography

[1. DeVerna, M. R. *et al.* CoVaxxy: A collection of English-language Twitter posts about COVID-19 vaccines. *In Proc. Intl. AAAI Conf. on Web and Social Media (ICWSM), 2021.* Preprint arXiv: 2101.07694. (2021).](https://www.zotero.org/google-docs/?QAUQgu)

[2. Towns, J. *et al.* XSEDE: Accelerating Scientific Discovery. *Comput. Sci. Eng.* **16**, 62–74 (2014).](https://www.zotero.org/google-docs/?QAUQgu)

[3. DeVerna, M. R. *et al.* Data for CoVaxxy: A collection of English-language Twitter posts about COVID-19 vaccines. https://github.com/osome-iu/CoVaxxy (2021).](https://www.zotero.org/google-docs/?QAUQgu)

[4. Dredze, M., Paul, M. J., Bergsma, S. & Tran, H. *Carmen: A Twitter Geolocation System with Applications to Public Health*.](https://www.zotero.org/google-docs/?QAUQgu)

[5. Lazer, D. M. J. *et al.* The science of fake news. *Science* **359**, 1094–1096 (2018).](https://www.zotero.org/google-docs/?QAUQgu)

[6. Shao, C. *et al.* The spread of low-credibility content by social bots. *Nat. Commun.* **9**, 4787 (2018).](https://www.zotero.org/google-docs/?QAUQgu)

[7. Grinberg, N., Joseph, K., Friedland, L., Swire-Thompson, B. & Lazer, D. Fake news on Twitter during the 2016 U.S. presidential election. *Science* **363**, 374–378 (2019).](https://www.zotero.org/google-docs/?QAUQgu)

[8. Pennycook, G. & Rand, D. G. Fighting misinformation on social media using crowdsourced judgments of news source quality. *Proc. Natl. Acad. Sci.* **116**, 2521–2526 (2019).](https://www.zotero.org/google-docs/?QAUQgu)

[9. Bovet, A. & Makse, H. A. Influence of fake news in Twitter during the 2016 US presidential election. *Nat. Commun.* **10**, 7 (2019).](https://www.zotero.org/google-docs/?QAUQgu)

[10. Golding, B. Iffy+ Mis/Disinfo Sites. *Iffy.news* https://iffy.news/iffy-plus/ (2020).](https://www.zotero.org/google-docs/?QAUQgu)

[11. MIT Election Data and Science Lab. U.S. President 1976–2020. (2021) doi:10.7910/DVN/42MVDX.](https://www.zotero.org/google-docs/?QAUQgu)

[12. Farrow, D. C., Brooks, L. C., Rumack, A., Tibshirani, R. J. & Rosenfeld, R. Delphi Epidata API. https://github.com/cmu-delphi/delphi-epidata (2015).](https://www.zotero.org/google-docs/?QAUQgu)
